# Quantifying the impact of addressing data challenges in prediction of length of stay

**DOI:** 10.1186/s12911-021-01660-1

**Published:** 2021-10-30

**Authors:** Amin Naemi, Thomas Schmidt, Marjan Mansourvar, Ali Ebrahimi, Uffe Kock Wiil

**Affiliations:** 1 Center for Health Informatics and Technology, The Maersk Mc-Kinney Institute, University of Southern Denmark, Odense, Denmark; 2 Department of Mathematics and Computer Science (IMADA), University of Southern Denmark, Odense, Denmark

**Keywords:** Length of stay, LOS, Classification, Regression, Machine learning, Vital signs, Health informatics, Emergency department, Data skewness

## Abstract

**Background:**

Prediction of length of stay (LOS) at admission time can provide physicians and nurses insight into the illness severity of patients and aid them in avoiding adverse events and clinical deterioration. It also assists hospitals with more effectively managing their resources and manpower.

**Methods:**

In this field of research, there are some important challenges, such as missing values and LOS data skewness. Moreover, various studies use a binary classification which puts a wide range of patients with different conditions into one category. To address these shortcomings, first multivariate imputation techniques are applied to fill incomplete records, then two proper resampling techniques, namely Borderline-SMOTE and SMOGN, are applied to address data skewness in the classification and regression domains, respectively. Finally, machine learning (ML) techniques including neural networks, extreme gradient boosting, random forest, support vector machine, and decision tree are implemented for both approaches to predict LOS of patients admitted to the Emergency Department of Odense University Hospital between June 2018 and April 2019. The ML models are developed based on data obtained from patients at admission time, including pulse rate, arterial blood oxygen saturation, respiratory rate, systolic blood pressure, triage category, arrival ICD-10 codes, age, and gender.

**Results:**

The performance of predictive models before and after addressing missing values and data skewness is evaluated using four evaluation metrics namely receiver operating characteristic, area under the curve (AUC), R-squared score (R^2^), and normalized root mean square error (NRMSE). Results show that the performance of predictive models is improved on average by 15.75% for AUC, 32.19% for R^2^ score, and 11.32% for NRMSE after addressing the mentioned challenges. Moreover, our results indicate that there is a relationship between the missing values rate, data skewness, and illness severity of patients, so it is clinically essential to take incomplete records of patients into account and apply proper solutions for interpolation of missing values.

**Conclusion:**

We propose a new method comprised of three stages: missing values imputation, data skewness handling, and building predictive models based on classification and regression approaches. Our results indicated that addressing these challenges in a proper way enhanced the performance of models significantly, which led to a more valid prediction of LOS.

## Background

Length of Stay (LOS) refers to the amount of time a patient spends in the hospital during a single visit, and it is regarded as one of the most important indicators for hospital resources utilization [1]. It has been shown that LOS is associated with other clinical outcomes, for example, LOS in an intensive care unit (ICU) exceeding three days is correlated with increased long-term mortality [2]. Lingsma et al. [3] also found that there is a relationship between LOS and mortality during the index admission, and Sud et al. [4] showed that long LOS is associated with increased rates of all types of mortality and readmission. It is worth noting that LOS is one of the simplest outcomes to calculate and extract, and since it relates other clinical outcomes, it can be used as a surrogate for other clinical outcomes e.g. in-hospital mortality as well as an index of patients’ illness severity [1].

The last decade has seen ongoing efforts to entice hospitals to adopt policy changes that improve efficiency by decreasing avoidable readmissions and patients’ LOS; it has been stated that 20% of all admissions are avoidable [5, 6]. This resulted in financial pressure and limited numbers of beds in hospitals, which made it crucial for hospital management to find solutions for cost reduction and optimal hospital resource usage [7]. Moreover, emergency department (ED) crowding has become one of the most important healthcare challenges in developed countries and interestingly, LOS can be both the reason and the result of crowding and associated with longer LOS [8–10]. Consequently, LOS reduction, admission rate reduction, and cost management have emerged as three of the most important issues for healthcare systems today [11].

Over the last few years, there has been a rapid growth in the utilization of machine learning (ML) techniques in healthcare domains, and the list of applications where ML matched or even outperformed clinicians has been expanding [12]. Thus, prediction of discharge time and LOS of each patient using ML and data mining can be one of the solutions to the abovementioned issues [13]. Moreover, by using proper and accurate predictive models, hospitals can avoid and control ED crowding, scale their capabilities over long-term strategic planning, and estimate healthcare costs. Also, a reasonable prediction of LOS as an initial assessment of patients’ illness severity is crucial for better resource allocation [14].

Various ML techniques, including logistic regression (LR), K-nearest neighbors (KNN), support vector machine (SVM), decision tree (DT), random forest (RF), and artificial neural networks (NN) have been utilized to predict the LOS of patients with different types of illnesses and clinical settings [5, 13, 15–17]. Combes et al. [18] implemented ML classifiers, including LR, SVM, DT, and RF to predict LOS of patients hospitalized at ED. Rahman et al. [19] developed a ML model based on DT for the prediction of prolonged LOS (LOS > 4 h) in ED. Barnes et al. [20] showed that ML models based on LR and RF for prediction of LOS based on admission data obtained in the ED outperformed hospital staff in terms of sensitivity and specificity. Some studies used regression models for prediction of LOS. For example, Caetano et al. [21] tested and compared regressors including multiple regression, DT, NN, SVM, and RF, where RF obtained the best performance. Turgeman et al. [22] applied several ML models and concluded that Cubist tree had the best prediction performance in their settings. They also stated that one of the advantages of tree-based models is that the learned rules are interpretable by humans.

Although different ML models have been developed to predict patients’ LOS, there are still certain issues that require more investigation. First, most research in this area have ignored important challenges such as dealing with missing values, and LOS skewed distribution. One of the most significant challenges in most datasets, particularly electronice health records (EHR), is the presence of missing values [23]. Most studies removed incomplete records or used statistics such as median to replace missing values. Some current studies in this domain do not provide information about the way that they dealt with incomplete records [5, 16–18, 20]. Other approaches for addressing the missing values problem include substitution of missing values of a patient by mean value from data of that patient [13], using stable patients’ information [15], and using specific characters (such as ‘NA’) for missing values [19]. These techniques ignore the correlation between variables, and this can have an adverse effect on the performance of predictive models. Considering complete case analysis results in significantly smaller datasets and lower statistical expressiveness for medical datasets. Using simple statistics or using a special value for missing variables does not generate useful information to improve model performance. It has been shown that, under the assumption of randomness of occurring missing values, using multivariate imputation techniques has substantial advantages that lead to statistically valid and unbiased results. It also provides beneficial information to enhance predictive models performance [24]. In addition, patients with missing values might have valuable information that improves the performance of predictive models [25]. A study by Bech et al. [26] concluded that one of the reasons for missing values in vital signs at arrival is that clinicians can recognize patients in a poor clinical condition at arrival without measuring and registering vital signs. They also showed that missing values in all variables are associated with short-term mortality. Therefore, samples with missing values can be informative and probably identify patients with severe conditions, so it is essential to take incomplete records into account and apply proper techniques to interpolate the missing values.

Another issue is data skewness, that can lead to imbalanced data and could be the result of two factors: (1) non-uniform sampling over the target variable, and (2) scarce evidence of the desired outcome among the samples. In skewed data, the tail region may act as an outlier for the ML models and adversely affect the models’ performance, especially regression-based models. Nevertheless, most current studies ignored the fact that LOS is typically highly skewed [22]. Carter et al. [5] have criticized most studies in this domain for neglecting the skewness of LOS data. Moreover, many studies have simplified the prediction problem to a binary classification (long LOS versus short LOS). In our previous study [27], we used patients’ arrival vital signs, age, and gender and stratified admitted patients into five classes based on their LOS. A multivariate ML-based imputation technique and a proper resampling technique were implemented to address missing values and data skewness challenges, respectively. Our results showed that addressing these challenges, which were ignored by many studies, can improve the prediction performance of all ML models.

### Objectives and contributions

In this study, we investigate the impact of data challenges including missing values and data skewness on the performance of ML models for prediction of LOS at ED, and how addressing these issues improves the results. To have a better estimation, both classification and regression approaches are implemented. The association between missing values and clinical deterioration is also evaluated. Therefore, the contributions of this study are threefold: (1) We have added more features to those we used previously, including patient arrival ICD-10 codes and triage categories. This richer dataset improved the models’ performance by an average of around 3% over our previous study, that used a classification approach. (2) In this study, we also show that there is a relationship between patients who are at severe risk of experiencing adverse events, missing values and data skewness, and discuss why solving missing values and data skewness is clinically important. (3) Instead of using limited number of classes, a regression approach that can provide more accurate results, is implemented and the impact of fixing the abovementioned challenges on the models’ performance is evaluated.

## Materials and method

### Study design and cohort selection

This study was designed based on transparent reporting of a multivariable prediction model for individual prognosis or diagnosis (TRIPOD) statement [28]. The data was collected during a cluster-randomized trial (CRT) of a novel patient dashboard. During the CRT, we collected vital signs including pulse rate (PR), respiration rate (RR), arterial blood oxygen saturation (SpO2), and systolic blood pressure (SBP) data from all hospitalized patients admitted to the ED of Odense University Hospital (OUH) between June 2018 and April 2019. Data was gathered from two sources: (1) the Philips IntelliVue patient monitors through a HL7 interface and registered by the dashboard backend, and (2) the clinical logistics system in use at ED [29]. Other patient information such as gender, age, admission and discharge dates, arrival ICD-10 codes of patients, and initial assessment of patients by clinicians using the adaptive process triage (ADAPT) system was collected through EHR data and stored in the dataset. Based on patients’ complaints and vital signs, ADAPT divides patients into four coded categories. Among these two variables, the more critical triage group specifies the final color-coded group for each patient. These groups are *Red*, *Orange*, *Yellow*, and *Green*. *Red* denotes more critical circumstances that require a greater priority, while *Green* denotes less urgent problems that may be addressed with a lower priority [30]. Table 1 shows the ADAPT scoring system.

**Table 1:**
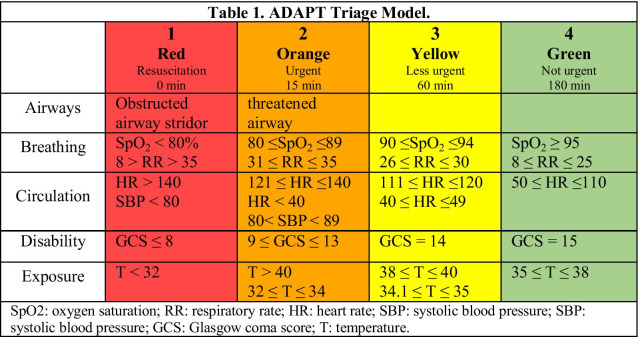
ADAPT triage model

Personal identification numbers were omitted from the database since they are classified as confidential data. All data was maintained on secure GDPR compliant servers hosed by the Open Patient data Explorative Network (OPEN)^1^ at the Region of Southern Denmark.

### Outcomes

The outcomes of this study are defined as: clinical deterioration includes receiving aid from the ICU, transfer to the ICU, and mortality, and LOS is also calculated by subtraction of admission date and time from the discharge date and time.

### Missing values imputation

In this study, a vital sign variable for a patient was defined as missing when there was no measurement for that patient’s vital sign during admission at all. In this study, multivariate imputation techniques, that impute missing values of a variable according to that variable and its correlation with other variables, were implemented. Six ML-based imputers, including KNN, DT, Gradient Boosting (GB), RF, Bayesian Ridge (BR), and Gaussian Process (GP) were used and a brief description for each technique is provided in Table 2.

**Table 2:** Summary of implemented ML algorithms

Algorithm	Description
KNN [31]	KNN is an instance-based and supervised ML algorithm used in both classification and regression problems. This algorithm uses *feature similarity* to determine the values of unseen data samples, meaning a missing datapoint is assigned a value based on that datapoint’s value in similar samples in the training set
BR [31]	BR is a linear regression using probability distributions rather than point estimates. In other words, the target variable’s value is not estimated as a single point but is drawn from a probability distribution
DT [31]	DT is a supervised learning model used in classification and regression applications that predicts the target value by learning simple rules from features. Each internal node represents a test on a feature, every leaf node indicates label of a class, and branching reflects features conjunctions that result in those classes
SVM (SVR) [31]	SVM is an instance-based and supervised ML algorithm utilized in classification and regression problems. A version of SVM for regression is called SVR. This algorithm generates a boundary known as a hyperplane between classes. The primary goal of this model is to maximize separation between classes
RF [32]	RF is a form of ensemble learning which is based on bagging. RF can be applied in both classification and regression applications. Ensemble learning refers to the process of constructing a model by merging various single models to produce a model that outperforms the primary models. In this approach, multiple subsets of a dataset are selected randomly with replacement, and one model is trained for each subset of the dataset. Ensemble learning has benefits such as overfitting prevention, solving the curse of dimensionality problem, and avoiding local optimal
GB [32]	GB is a supervised ensemble learning algorithm based on boosting that can be applied in both classification and regression applications. In this approach, basic models are trained sequentially which means the output of each model passes to the next model
XGB [32]	XGB is an enhanced version of GB and both techniques use the gradient boosting principle. Differences in details of the modeling process include a more regularized way of formalization to avoid over-fitting and generally provide better performance
GP [31]	GP is a stochastic process that consists of random variables and every finite set of random variables is modeled by a multivariate normal distribution. GP is a supervised learning technique that can be used for both classification and regression problems
NN [31]	NN is a supervised ML algorithm that has single or multiple layers between input and output layers called hidden layers that carry data from input to output layer. NN can be implemented in classification and regression applications. In this study, a feedforward architecture was utilized

KNN, K-neasrest neighbors; BR, Bayesian ridge; DT, decision tree; SVM, support vector machine; SVR, support vector regression; RF, random forest; GB, gradient boosting; XGB, extreme gradient boosting; GP, Gaussian processing; NN, neural network

The imputers were evaluated using complete records without missing values, and fivefold cross-validation was used to achieve a better estimation about the performance of models. At each iteration, 25% of the training set was used for validation. Grid search was applied to find the optimal hyperparameters and based on that, the number of neighbors for KNN was 10, number of iterations for iterative models was 100, mean square error was used as loss functions and criterion, number of DT for RF and GB was 50, the max depth for DT, RF, and GB was 30, and normal distribution with mean 0 and standard deviation 1 was used for GP.

### Data imbalance

As mentioned in “Background” section, LOS data distribution is usually highly skewed [22], and most LOS prediction studies have ignored this challenge. In this study, resampling techniques were applied to overcome the data skewness challenge. Undersampling and oversampling are the two main approaches for resampling. In undersampling, the number of records in the majority class is decreased, while oversampling increases the minority class samples. Two following sections introduce proper techniques implemented for solving data imbalance for classification and regression approaches.

#### Classification approach

One of the most appropriate methods for dealing with the class imbalance issue is the synthetic minority oversampling technique (SMOTE) [33]. SMOTE chooses close samples in the feature space, draws a line between them, and synthesizes new samples along that line. One limitation of this technique is that synthetic samples are created without taking the majority class into account, which can result in ambiguous samples if the classes have high overlap. Several extensions of SMOTE, such as Borderline-SMOTE [34], have been developed to reduce this ambiguity. Borderline-SMOTE focuses on the difficult instances by providing more resolution where needed. In other words, instead of oversampling all samples of the minority class, Borderline-SMOTE focusses on oversampling minority class samples that are close to the borderline.

#### Regression approach

The data imbalance challenge has been studied extensively in the context of classification applications, but this issue can also occur in other domains, such as regression. However, suitable strategies for other domains are scarce and only a few algorithms have been proposed, such as SMOTER (an extension of SMOTE) and the introduction of Gaussian noise (IGN). Branco et al. [35] proposed a technique called SMOGN that combines both abovementioned techniques to reduce limitations of both techniques. In other words, by using SMOGN: 1) the risk of using SMOTER is reduced because SMOTER does not consider distant examples during the interpolation process; and 2) generalization power increases, allowing the decision boundaries to be extended for the rare cases.

### Predictive models development

Following the data preparation phase, ML models were developed to predict the LOS of patients. We used data collected from patients at arrival time, including PR, RR, SpO2, SBP, ICD-10 codes, ADAPT triage categories, age, and gender. There are 723 unique ICD-10 codes in the dataset, however, 617 of them had a frequency of less than 10 repetitions among patients. For the sake of keeping the generalization power of predictive models as well as avoiding the *curse of dimensionality*, only the first three letters of ICD-10 codes were used as features. According to this, with a fixed number of training samples, the predictive performance a regressor or classifier initially improves when the number of dimensions (features) increases, however, after a certain dimensionality it begins to deteriorate rather than improve steadily [31]. The ICD-10 codes were mapped using one hot encoding before feeding to the ML models. Five ML algorithms for both classification and regression approaches were applied, i.e., NN, XGB, RF, SVM (SVR for regression), and DT. A summary of these ML models is presented in Table 2. fivefold cross-validation and grid search were applied to determine the optimal models’ hyperparameters. The number of neurons for each layer of NN in classification and regression approaches were (three hidden layers: 133, 64, 32, 8, 5), and (four hidden layers: 133, 64, 32, 8, 4, 1), respectively. For NN training 1000 epochs and Adam training algorithm were used. Number of DT in RF and XGB were 50, and maximum depth for DT, RF, and XGB was 30. Parameters of SVM were Kernel = RBF, C = 1, and gamma = 0.001. Analysis was performed using Python 3.7.7 and scikit-learn and TensorFlow libraries.

### Predictive models evaluation

Four metrics including Receiver operating characteristics (ROC) curve and area under the curve (AUC) for classification, and R-squared score (R^2^) and Normalized Root Mean Square Error (NRMSE) for regression were used. ROC is a reliable metric that represents the sensitivity over all possible values of specificity or vice versa, and AUC is an effective metric for summarizing the accuracy of models. In the regression approach, the R^2^ score is a relative measure of fit and shows how well the predictive models fit the dependent variable, whereas NRMSE indicates the absolute fit of the models to the data and indicates how close observed samples are to the predicted values. We evaluated the performance of predictive models at three stages (original data, imputed data, imputed and resampled data) to investigate the impact of missing values imputation and resampling on the performance of models. R^2^ score and NRMSE are calculated as follow,

1 $$\begin{aligned} R^{2} & = 1 - \frac{{\mathop \sum \nolimits_{i} \left( {LOS_{i} - \widehat{LOS}_{i} } \right)^{2} }}{{\mathop \sum \nolimits_{i} \left( {LOS_{i} - \overline{LOS} } \right)^{2} }} \\ NRMSE & = \frac{{\sqrt {\frac{{\mathop \sum \nolimits_{i} \left( {LOS_{i} - \widehat{LOS}_{i} } \right)^{2} }}{n}} }}{{LOS_{max} }} \\ \end{aligned}$$

where ${LOS}_{i}$ and ${\widehat{LOS}}_{i}$ are the real LOS and the predicted LOS of patient number $i$, respectively. $\overline{LOS }$ denotes the average of LOS for all patients and *n* is the total number of patients and ${LOS}_{max}$ is the maximum LOS among all patients. In the regression approach, there is no rule of thumb for good values for R^2^ and it varies based on different applications, however, having a high R^2^ for an application based on its literature and small NRMSE represents good results.

## Results

47,492 visits to the OUH have been registered. After removing not hospitalized records, under 18 years old records, records with no information about arrival status, 6027 records were selected as the dataset. Of these 6207 patients, 101 patients deteriorated during hospitalization. Table 3 summarizes statistics of deteriorated and not-deteriorated groups of patients.

**Table 3:** Data description

	Not deteriorated patients (n = 5926)		Deteriorated patients (n = 101)	
	Statistics	Missing ratio (%)	Statistics	Missing ratio (%)
Gender, Male n (%)	3195 (53%)	0	54 (54%)	0
Age, Median (IQR)	68 (52–80)	0	78 (70–88)	0
PR (min^−1^), Median (IQR)	83 (69–99)	2.45	94 (81–103)	2.11
RR (min^−1^), Median (IQR)	18 (14–22)	3.01	24 (16–33)	14.36
SpO2 (%), Median (IQR)	96 (92–99)	2.35	92 (81–99)	2.51
SBP (mmHg), Median (IQR)	129 (110–147)	7.78	100 (52–160)	10.28

min, minute; PR, pulse rate; IQR, interquartile range; RR, respiratory rate; SpO2, oxygen saturation; mmHg, millimeter of mercury; SBP, systolic blood pressure

**Fig. 1:**
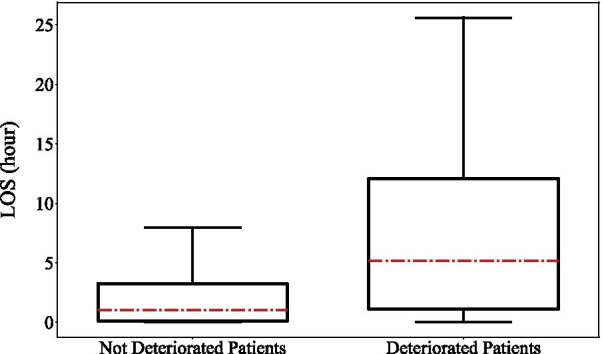
Boxplots of LOS for not deteriorated and deteriorated patients

LOS of deteriorated and not deteriorated patients was investigated using Kruskal test with statistically significant level as *p* < 0.05. The result (Fig. 1) indicated that there was a statistical difference between not deteriorated and deteriorated patients (*p* < 0.0001), and deteriorated patients had significantly longer LOS.

**Fig. 2:**
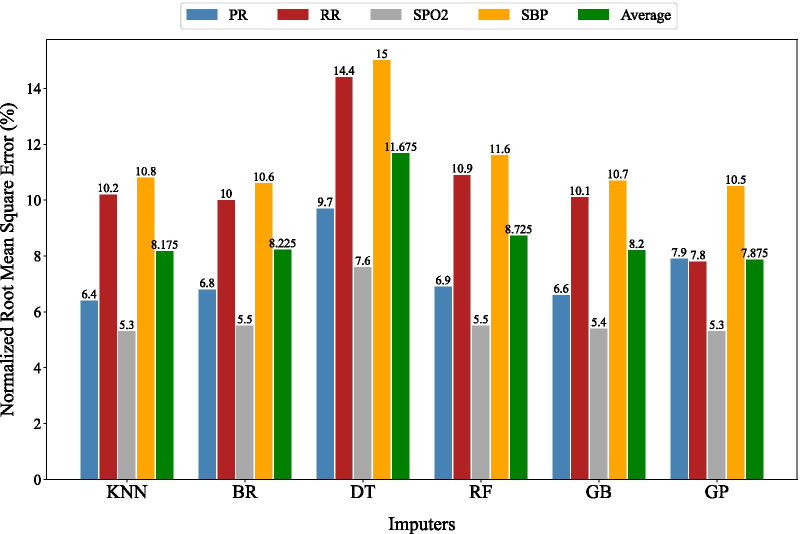
Imputation models performance based on NRMSE

The best approach for imputation was determined by analyzing the information from the 5331 patients with no missing values. NRMSE as shown in Eq. (1) was used as the evaluation metric and the results are presented in Fig. 2. As seen, GP, with around 92% accuracy on average, produced superior performance than other models, so it was used for the imputation of missing values in 696 dataset entries with missing values.

### Classification approach

**Fig. 3:**
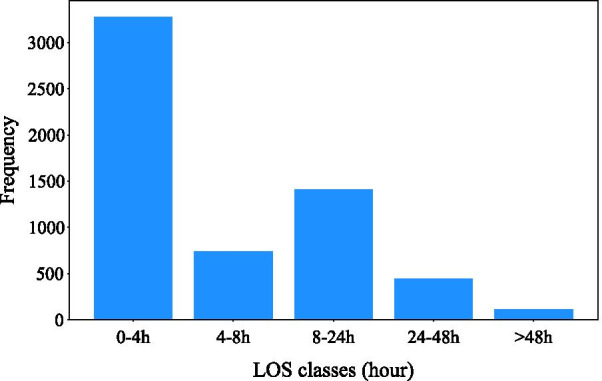
LOS distribution by hour (classification approach)

Patients were grouped into five groups as (0–4 h), (4–8 h), (8–24 h), (24–48 h), and (> 48 h) based on clinical guidelines in the ED. Figure 3 shows the LOS distribution by hour.

**Fig. 4:**
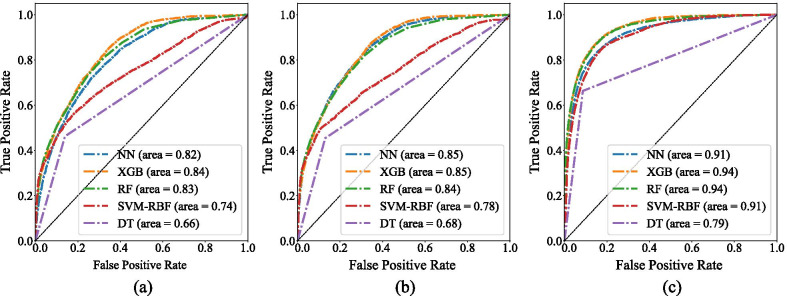
Performance of predictive models for classification approach: **a** on original data, **b** after applying GP imputation and before applying Borderline-SMOTE, **c** after applying GP imputation and Borderline-SMOTE

Figure 3 shows that the distribution is highly skewed, so Borderline-SMOTE was used to increase the number of samples in the minority classes to ensure that these classes were also learned adequately. ROC and AUC were used as evaluation metrics and the performance of models was assessed before and after applying GP imputer and Borderline-SMOTE. The results are displayed in Fig. 4. As shown, interpolation of missing values (Fig. 4b) and addressing data imbalance problems (Fig. 4c) enhanced the performance of all predictive models.

### Regression approach

**Fig. 5:**
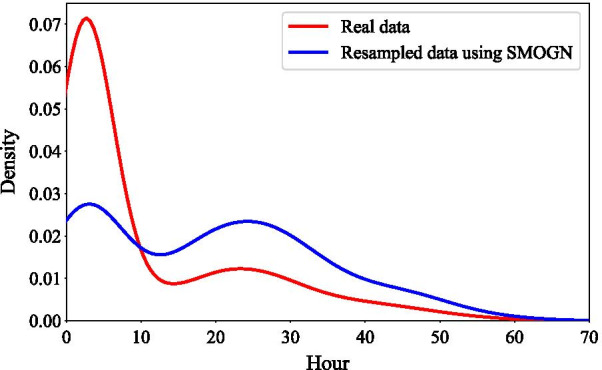
Distribution of LOS by hour (regression approach)

One of the limitations of using the classification approach is that a wide variety of patients are categorized into a limited number of classes. A regression approach was used to address this limitation, where actual LOS is recorded as a continuous variable in hours, (shown as a red curve in Fig. 5). The overall distribution can be seen to be similar to Fig. 3 and similarly skewed. Therefore, the SMOGN resampling technique was applied to achieve more balanced samples for training; the blue curve shows the resampled dataset using SMOGN. Based on excess Kurtosis that quantifies the tail characteristics of a distribution, the excess Kurtosis value for the data changed from 1.45 to -0.75 after resampling, where the negative excess Kurtosis indicates that the samples located at the tail of distribution in the resampled dataset are less extreme than the actual dataset. In other words, in the resampled dataset, there is more evidence for samples placed on the right side of distribution and far from the mean. Consequently, ML models have more information about patients with longer LOS and do not underpredict these patients, and the results are more reliable.

**Fig. 6:**
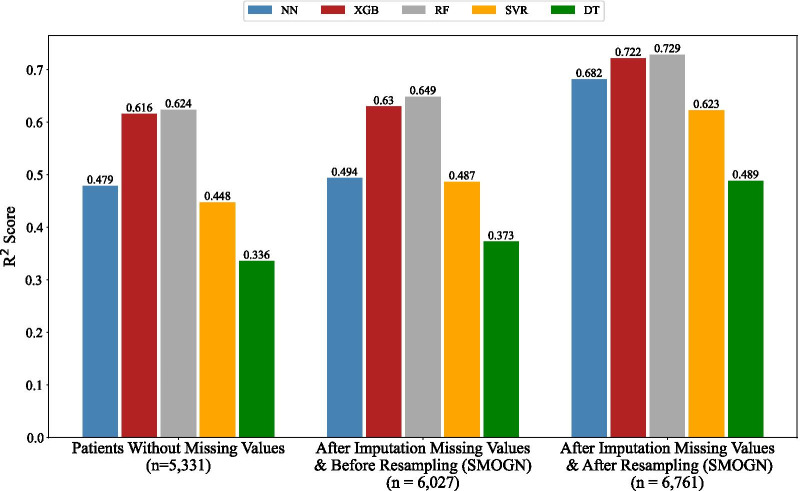
Performance of ML predictive models in regression approach based on R^2^ score

The performance of ML models in prediction of LOS in hours based on R^2^ score is presented in Fig. 6. As seen, all ML models had the best performance for the imputed and resampled data.

**Fig. 7:**
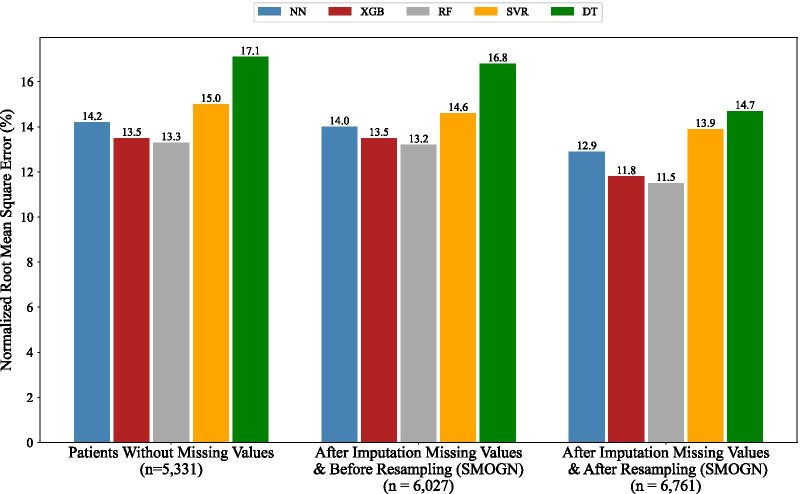
Performance of ML predictive models in regression approach based on NRMSE

Moreover, the performance of ML models based on NRMSE was also investigated and the results is shown in Fig. 7. As seen, error rate of all ML models was less for imputed and resampled data. Figures 6 and 7 indicate that addressing missing values and data skewness had a constructive impact on the performance of all ML regressors in prediction of LOS.

**Fig. 8:**
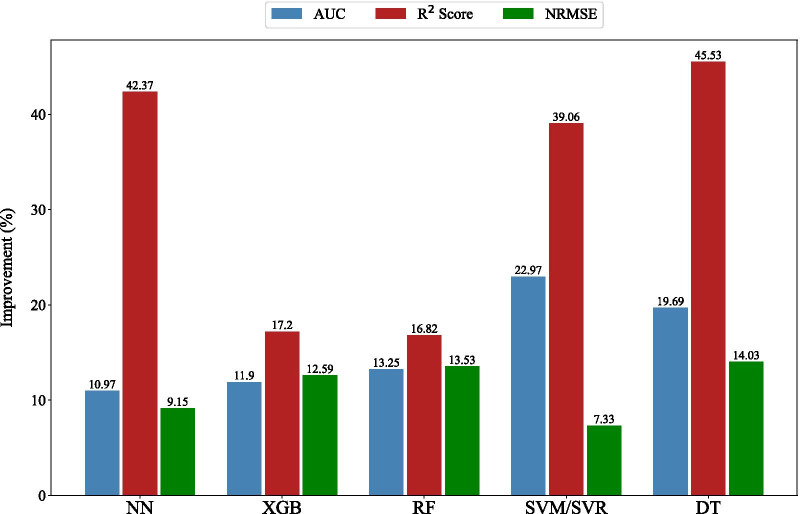
Improvement of ML models’ performance based on Eq. (2)

To quantify the improvement of models’ performance in predicting the real LOS for both classification and regression approaches, Eq. (2) was used, and the results is displayed in Fig. 8.

2 $$Improvement = \frac{{\left| {Performance_{original } - Performance _{imputed\,and\,resampled\,data} } \right|}}{{Performance_{original } }} \times 100$$

Based on Fig. 8, performance of ML models in both approaches improved. Specifically, for all models, R^2^ score had the most improvement among three metrics and for three models (NN, SVM/SVR, DT), the R^2^ score enhanced significantly.

## Discussion

It has been stated that LOS is a clinical outcome that can be used as a surrogate for other outcomes such as severity of patients’ problems. Research has shown that prolonged LOS is associated with higher patient mortality and morbidity [36, 37], hence identifying patients with longer LOS can be used as an alternative way for early detection of clinical deterioration. It has been also stated that understanding characteristics of patients with longer LOS enables us to develop more effective models, plan hospital resources more efficiently, and reduce unnecessary procedures at hospitals [38, 39]. Our results confirmed previous findings that patients at greater risk of deterioration stay in hospital longer (have a much higher LOS). Moreover, a recent study [26] has shown that there is an association between missing values and illness severity of patients, meaning that patients in a bad clinical condition at arrival are more likely to have missing values in their collected records which our results also confirmed this finding and showed a relatively higher rate of missing values for deteriorated patients (Table 3). However, the low representation of this group of patients in datasets collected at EDs creates challenges for training predictive models and leads to a decline in their performance. First, most people admitted to an ED recover well so the average rate of patients who deteriorate is only around 2% of all hospitalized ED patients [40]; second, most of these patients have longer LOS than average and since the LOS data distribution is left-skewed, the data entries acquired for this type of patients is rare and they cannot contribute to tuning models effectively during the ML training phase. Finally, based on [26] and our findings, these patients are more likely to a have higher rate of missing values, so by removing incomplete records or using improper imputation techniques in analysis, the information about these patients is reduced even more.

These challenges prevent us to understand the characteristics of patients. It is imperative to increase the data from different types of patients in clinical datasets to have a fair and reliable prediction that can help clinicians and hospitals to detect different kinds of patients and assess their needs, especially for those who are at the risk of adverse events. Interestingly, previous studies indicated that the missing values in patients’ records can be addressed properly by using multivariate imputation techniques [24, 26, 41] that can have a significant impact on the performance of ML models in predicting adverse events [25] that lead to early intervention; as a result it, decreases mortality rate by in time responding to deteriorating patients [42, 43].

It has been estimated that up to 80% of data analysis time is dedicated to data preparation and this phase is crucial for building successful ML predictive models [32]. Therefore, in this study, we considered two data challenges including missing values and data skewness, and presented an effective way for imputation, following by resampling techniques that produced synthetic records for patients with longer LOS. The final parts of our study were ML models development and quantifying the impact of addressing the abovementioned challenges in predicting LOS at ED. Most of the current studies were based on binary classification and used thresholds to group patients [19, 39, 44–47], but we know that patients need different levels of care and grouping them into two classes is insufficient as a wide variety of patients are placed in one group; resulting in ML models that cannot simulate the condition of more severe patients and underprediction of their LOS. To overcome this challenge, in the first step we increased the number of classes to five, then we go further and developed regression models that predict patients’ LOS in hours. Various ML models have been used in different studies to predict LOS; however it has been demonstrated that no model fulfill all of the requirements for predicting LOS [33]. Thus, in this study for having a comprehensive estimation, both classification and regression approaches and several ML models with different theoretical backgrounds and capabilities were implemented. To investigate the effect of resampling on the estimation accuracy of patients with higher LOS, we checked the NRMSE of prediction by RF on the fourth group of patients (LOS = 24–48 h) who were likely to be relatively severe patients. The NRMSE for this group before and after resampling was 0.388 and 0.186 which indicated that after resampling, RF performed more accurately, presumably due to having more complete data on the patients. Implementing the LOS as a continuous variable instead of five classes also led to more accurate prediction however, it needs more data samples.

Our results highlighted the importance of the two preprocessing steps and their impacts on the performance of predictive models, especially in the regression case when the task is more complicated and sensitive to the challenges of skewed data and missing values. Our solutions improved the performance of ML predictive models by an average of 15.75% (AUC metric), 32.19% (R^2^ score metric), and 11.32% (NRMSE metric) for classification and regression domains, respectively (Fig. 8). It is worth mentioning that we had remarkable improvement in the performance of some models which are more dependent on the number of samples and their representativeness, e.g. performance of NN in regression domain increased around 42% in terms of R^2^ score. Based on Mandrekar’s suggestion [48], the performance of three models (NN, RF, XGB) changed from excellent to outstanding; SVM changed from acceptable to outstanding, and DT reached acceptable performance.

## Strengths and limitations

Our study has several strengths. We used information of unspecific ED patients which means heterogenous patients are considered in our analysis. Moreover, we evaluated several ML techniques and approaches for missing values imputation and building predictive models. However, our study has some limitations that should be recognized. As stated in this study, we collected our dataset from one source, Odense University Hospital, which limits the generalizability of results. Therefore, it is essential to investigate the performance of proposed solutions and validate them using other ED datasets. Although we implemented models such as RF that produces interpretable results, clinical applicability of models should be evaluated using prospective study. Moreover, this study was conducted using data collected at an ED, however there might be other challenges or patterns in other clinical environments such as ICU. Therefore, it is recommended to evaluate findings of this study in other clinical settings.

## Implication

The primary audience for this study are researchers that intend to perform secondary analysis using EHR datasets. As shown, data challenges such as missing values, noise, sparseness, and messiness are undeniable issues in EHR datasets [23, 49], thus addressing these challenges is essential work before conducting any analysis. Our models can be used as an auxiliary tool helping clinicians assess patients’ conditions to enable in time interventions. Our findings can also aid hospitals to better plan their resources. ML experts and developers interested in developing models or decision support systems for predicting LOS or clinical deterioration can also utilize the findings of this study to assess different techniques and select appropriate models for prediction tasks.

## Conclusion

This study investigated important challenges in prediction of LOS, presented solutions for them, and measured the impact of these solutions on the performance of predictive models using both classification and regression approaches. The results demonstrated that addressing these challenges improved the performance of all ML models, especially in the regression domain, where these challenges reduce models’ performance significantly. Moreover, the results showed that more severe patients had a relatively higher rate of missing values and fewer complete entries in the dataset, so ignoring incomplete records or interpolating them in an inappropriate way leads to underprediction of the more sensitive and challenging groups of patients.

Ignoring data skewness in particular may lead to under-prediction of LOS and unreliable results. The ultimate goal of LOS prediction, especially at the admission time, is to help hospital management to allocate and plan their resources, to help clinicians obtain reliable insight into patients’ conditions, and possibly use LOS as an index for assessing the severity of patients. In this regard, patients with longer LOS represent the biggest challenge, as this group most substantially affects both prediction accuracy and hospital resource planning and can result in ED overcrowding. Unfortunately, these patients are usually underrepresented in the raw datasets, so statistically increasing the proportion of these patients plays a key role in the performance of predictive models. Therefore, recognizing these potential challenges in the data and solving them effectively is vital for building effective ML models. Our work has illustrated the importance of this, and we recommend researchers pay more attention to data preprocessing to produce robust methods that avoid bias and under-prediction.

## Data Availability

The dataset used for this study is not publicly available due to possible compromising individual privacy but is available from the corresponding author on reasonable request.

## Abbreviations

LOS: Length of stay
ED: Emergency departments
ML: Machine learning
LR: Logistic regression
KNN: K-nearest neighbors
SVM: Support vector machine
DT: Decision tree
RF: Random forest
NN: Artificial neural networks
GP: Gaussian process
XGB: Extreme gradient boosting
SVR: Support vector regression
OUH: Odense University Hospital
SpO2: Arterial blood oxygen saturation
PR: Pulse rate
RR: Respiration
SBP: Systolic blood pressure
ADAPT: Adaptive process triage
OPEN: Open patient data explorative network
EHR: Electronic health records
GB: Gradient boosting
BR: Bayesian ridge
SMOTE: Synthetic minority oversampling technique
IGN: Introduction of Gaussian noise
ICU: Intensive care unit
NRMSE: Normalized root mean square error
ROC: Receiver operating characteristics curve
AUC: Area under the curve

## Footnotes

1 https://open.rsyd.dk.
